# Mammalian cell entry operons; novel and major subset candidates for diagnostics with special reference to *Mycobacterium avium* subspecies *paratuberculosis* infection 

**DOI:** 10.1080/01652176.2019.1641764

**Published:** 2019-07-08

**Authors:** Zahra Hemati, Abdollah Derakhshandeh, Masoud Haghkhah, Kundan Kumar Chaubey, Saurabh Gupta, Manju Singh, Shoorvir V. Singh, Kuldeep Dhama

**Affiliations:** a Department of Pathobiology, School of Veterinary Medicine, Shiraz University, Shiraz, Iran;; b Department of Biotechnology, Institute of Applied Sciences and Humanities, GLA University, Mathura, India;; c Department of Pathology, Indian Veterinary Research Institute, Bareilly, India

**Keywords:** *mce* operon, Mycobacteria, protein, diagnosis, MAP, MTB, Johne’s disease

## Abstract

Mammalian cell entry (*mce*) genes are the components of the *mce* operon and play a vital role in the entry of Mycobacteria into the mammalian cell and their survival within phagocytes and epithelial cells. *Mce* operons are present in the DNA of Mycobacteria and translate proteins associated with the invasion and long-term existence of these pathogens in macrophages. The exact mechanism of action of *mce* genes and their functions are not clear yet. However, with the loss of these genes Mycobacteria lose their pathogenicity. *Mycobacterium avium* subspecies *paratuberculosis* (MAP), the etiological agent of Johne’s disease, is the cause of chronic enteritis of animals and significantly affects economic impact on the livestock industry. Since MAP is not inactivated during pasteurization, human population is continuously at the risk of getting exposed to MAP infection through consumption of dairy products. There is need for new candidate genes and/or proteins for developing improved diagnostic assays for the diagnosis of MAP infection and for the control of disease. Increasing evidences showed that expression of *mce* genes is important for the virulence of MAP. Whole-genome DNA microarray representing MAP revealed that there are 14 large sequence polymorphisms with LSPP12 being the most widely conserved MAP*-*specific region that included a cluster of six homologs of *mce*-family involved in lipid metabolism. On the other hand, LSP11 comprising part of *mce*2 operon was absent in MAP isolates. This review summarizes the advancement of research on *mce* genes of Mycobacteria with special reference to the MAP infection.

## 1. Introduction

Mammalian cell entry (*mce*) operons are present in the DNA of Mycobacteria and translate proteins associated with the invasion and long-term existence of this pathogen in macrophages (Mohn et al. 2008; Senaratne et al. 2008; Zhu et al. 2008; Zhang and Xie 2011; Rathor et al. 2013; Rodriguez et al. 2015). The *mce* genes are also present in other species like *Nocardia*, *Janibacter*, *Nocardiodes*, *Amycolatopsis* and *Streptomyces* (Table 1) as well as in Gram-negative bacteria and have also been found encoded in plant genomes. In Gram-negative bacteria, a 98 amino acid sub-region of ‘Mce-like’ protein domain as part of the inner membrane lipid-binding proteins (PF02470) are widely distributed (Casali and Riley 2007; Isom et al. 2017). An operon is a functional unit of DNA containing a cluster of genes under the control of a single promoter. The *mce* operon was first discovered while studying the entry of *Mycobacterium tuberculosis* (MTB) inside host non-phagocytic cells (Ahmad et al. 2005; Timms et al. 2015). Arruda et al. (1993) first reported that a DNA fragment of MTB, namely H37Ra, conferred on a nonpathogenic *Escherichia coli* the ability to enter macrophage cells and was termed as *mce* gene (Kumar et al. 2003; Marjanovic et al. 2010; Zhang and Xie 2011). A total of 45 vital cell surface (exposed) antigens of Mycobacteria have been listed in Table 2 of which 6 are *mce* proteins. Although *mce* genes have been reported in many bacterial species, these genes exist as operons in Mycobacteria only (Timms et al. 2015). The *mce* operons encode sets of invasion/adhesion like proteins all predicted to contain hydrophobic stretches or signal sequences near the N-terminus. Their location on the Mycobacterial cell surface is in line with the potential role of *mce* operons in mammalian cell invasion, hence regarded as important virulence attributes (Harboe et al. 2004; Ahmad et al. 2005; Gioffre et al. 2005; Semret et al. 2005; Rodriguez et al. 2015). As the *mce* genes are absent in the human genome, these genes might also be represented as ideal candidates for drug targets (Zhang and Xie 2011).

**Table 1.** Distribution of *mce* genes within the order *Actinomycetales*.

Suborder	Family	Species	*mce*	Source
*Corynebacterineae*	*Mycobacteria ceae*	*M. leprae* TN	6	UniProt
		*M. bovis* AF2122/97	18	UniProt
		MTB CDC1551	24	TIGR
		MTB H37Rv	24	TIGR
		*Mycobacterium paratuberculosis* K-10	48	UniProt
		*M. smegmatis* MC2 155	34	TIGR
		*Mycobacterium* sp. MCS	38	JGI
		*Mycobacterium* sp. KMS	38	JGI
		*Mycobacterium* sp. JLS	50	JGI
		*Mycobacterium flavescens* PYR-GCK	48	UniProt
		*Mycobacterium vanbaalenii* PYR-1	66	UniProt
	*Nocardiaceae*	*N. farcinica* IFM 10152	36	UniProt
*Micrococcineae*	*Intrasporangiaceae*	*Janibacter* sp. HTCC2649	6	NCBI
*Propionibacterineae*	*Nocardioidaceae*	*Nocardioides* sp. JS614	12	UniProt
*Pseudonocardineae*	*Pseudonocardiaceae*	*Amycolatopsis mediterranei*	6	Pfam
*Streptomycineae*	*Streptomycetaceae*	*Streptomyces avermitilis* MA-4680	6	UniProt
		*S. coelicolor* A3(2)	6	UniProt

**Table 2.** List of MAP cell surface proteins and their functions.

S. No.	Mycobacterium structural proteins	Functions
1	MAP2189	Mammalian cell entry proteins
2	MAP2190	
3	MAP2191	
4	MAP2192	
5	MAP2193	
6	MAP2194	
7	MAP3567	Hypothetical protein
8	MAP1508	Hypothetical protein
9	MAP 0047c	Lpp-LpqN family conserved in Mycobacteria ceae
10	MAP0209c	Protein potentially involved in peptidoglycan biosynthesis in MAP
11	MAP3936	Chaperonin GroEL
12	MAP4143	Elongation factor Tu
13	MAP3024c	HupB
14	MAP3651c	FadE3_2
15	MAP1997	Acyl carrier protein
16	MAP3968	Heparin-binding hemagglutinin adhesin-like protein
17	MAP1122	MIHF
18	MAP1589c	Alkylhydroperoxidase C
19	MAP1506	Hypothetical protein
20	MAP3362c	S-adenosyl-L-homocysteine hydrolase
21	MAP1519	Hypothetical protein
22	MAP2698c	DesA2 DesA2
23	MAP1998	3-oxoacyl-(acyl carrier protein) synthase II
24	MAP3840	Molecular chaperone DnaK
25	MAP4264	co-chaperonin GroES
26	MAP3693	Acetyl-CoA acetyltransferase
27	MAP1563c	Hypothetical protein
28	MAP0398c	Probable transcriptional regulatory protein
29	MAP0896	Succinyl-CoA synthetase subunit beta
30	MAP0966c	Hypothetical protein
31	MAP3033c	SerA
32	MAP3007	Hypothetical protein
33	MAP3188	FadE24
34	MAP0990	Phosphopyruvate hydratase
35	MAP1588c	AhpD
36	MAP1164	Glyceraldehyde-3-phosphate dehydrogenase
37	MAP1889c	Wag31
38	MAP4233	DNA-directed RNA polymerase subunit alpha
39	MAP4167	rpsC
40	MAP3061c	Probable electron transfer flavoprotein (beta-subunit) fixed
41	MAP2228	Hypothetical protein
42	MAP4233	DNA-directed RNA polymerase subunit alpha
43	MAP2453c	AtpH
44	MAP3005c	Hypothetical protein
45	MAP2280c	ATP-dependent Clp protease proteolytic subunit

This review summarizes advancements of research on *mce* genes of Mycobacteria with special reference to the *Mycobacterium avium* subspecies *paratuberculosis* (MAP), the cause of incurable granulomatous enteritis known as Johne’s disease (JD) in domestic livestock. Since MAP is not inactivated during pasteurization, human population is continuously at the risk of getting exposed to MAP infection through consumption of dairy products. MAP has also been associated with human disorders mainly of auto-immune nature (Faisal et al. 2013; Wang et al. 2014; Sechi and Dow 2015; Waddell et al., 2015; Chaubey et al. 2017; Gupta et al. 2017).

Objectives of this review are to enlighten the importance of Mce proteins in pathogenesis of Mycobacterial infections as well as facilitating the development of new candidates for Mycobacterial diagnostics. Current diagnostics for Mycobacterial infection have focused on the use of surface proteins as antigenic bio-markers of Mycobacterial species to diagnose the infection, which may be helpful in the control of disease (Li et al. 2005; Souza et al. 2011; Moigne and Mahana 2012; Chaubey et al. 2016). Our focus in this review is on the Mce proteins because (1) most of the Mycobacteria having Mce proteins, and (2) the information may be helpful in better understanding of the patho-biological and immunological significance of Mce proteins and their roles in the virulence of the pathogens belonging to Mycobacterial species.

## 2. The *mce* operon in MTB

MTB is an intracellular pathogen and reside inside the macrophages which is the vital constituents of the immune system (Mukhopadhyay and Balaji 2011). The mechanism of the entry and survival of MTB inside macrophages have been poorly understood earlier, but recent findings showed the presence of multiple cells-surface receptors that influence the entry of MTB into the macrophages: mannose receptors, complement receptors CR3b and CR1, Fc receptors, fibronectin receptor, scavenger receptors, and Mce proteins (Harboe et al. 2004; El-Shazly et al. 2007; Zhang et al. 2017).

**Figure 1.**
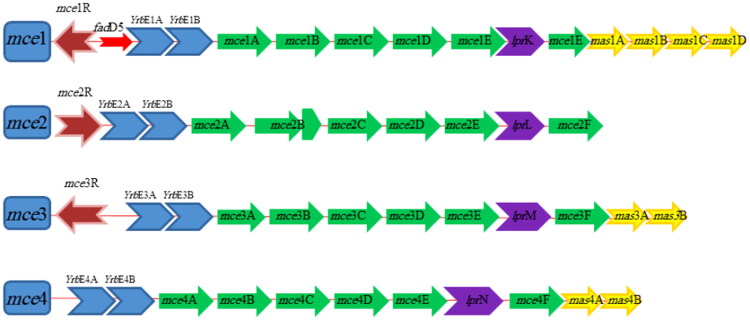
Schematic diagram of *M. tuberculosis mce* operons. Transcription regulators are colored in brown, *yrb*E genes in blue, *mce* genes in green, *mas* genes in yellow and genes encoding Mce-family lipoprotein (lpr) are shown in purple.

MTB genome possesses four dispersed, but homologous sets of genes called *mce* operons (*mce*1–*mce*4) organized in identical pattern and each *mce* operon translates into two integral membrane proteins (yrbEA-B) and six Mce proteins (MceA–F) (Ahmad et al. 2005; Uchiya et al. 2013). Hence, *mce* genes present in four operons and each operon is made up of eight genes (yrbEA-B and *mce*A–*mce*F) as shown in Figure 1. Differential expression of *mce*1–4 operons points toward their functional significance (Pasricha et al. 2011). Four downstream genes of MTB *mce*1 (Rv0175-78) operon, two downstream genes of MTB *mce*3 operon and two downstream genes of MTB *mce*4 operons are termed as ‘*mce*-associated proteins’ or Mas proteins, which are involved in Mce transporter function (Casali and Riley 2007) (Figure 1). Contribution of these *mce* genes on the pathogenicity of Mycobacteria may be determined by their level of expression (Haile et al. 2002; Marjanovic et al. 2010; Singh et al. 2016).

### 2.1. Mammalian cell entry 1 (mce1) operon

The *mce*1 operon is present in all species of Mycobacteria. MTB encodes six (*mce*1A–*mce*1F) invasion-like proteins that localize to the cell wall and are involved in substrate trafficking (Chitale et al. 2001; Shimono et al. 2003; Stavrum et al. 2012). The *mce*1A (Rv0169) operon helps to change of plasma membrane in host mammalian cells that promote the uptake of products bound to it (Chitale et al. 2001). The Mce1 proteins taking part in mycolic acid or fatty acids importation (Marjanovic et al. 2011; Forrellad et al. 2014) and glutamate and phosphatidic acid as possible substrates of the new Mce transporters (Dassa and Bouige 2001).

Purified recombinant Mce1A protein coated on latex beads are internalized by non-phagocytic HeLa cells (Chitale et al. 2001; Kumar et al. 2003). Further studies have shown that *mce*1A gene deletion in *M. bovis* BCG (Bacille Calmette-Guerin) decreases in bacterium ability to invade Hela cell (Gioffre et al. 2005; Obregon-Henao et al. 2011; Zhang and Xie 2011; Castellanos et al. 2012). Beste and colleagues (2009) opined similar scenario for MTB *mce*1 mutants and reported these mutants were unable to enter, or exit early from, the slow growth rate state and are thereby over represented in slow growth rate cultures. Additionally, the *mce*1 operon proteins are involved in mycolic acid recycling and fatty acid transport (Stavrum et al. 2012). Forrellad et al. (2014) also demonstrated that the lack of Mce1 proteins affects the uptake of fatty acids. In addition, Rv0165c gene (a putative transcriptional regulator) is localized upstream of *mce*1 operon and this Mce1R regulator facilitates the balanced expression of the Mce1 proteins that are important for granuloma formation, which is necessary for the perseverance of Mycobacteria (Gioffre et al. 2005; Zhang and Xie 2011). It has been shown that *mce*1R gene (GntR-negative transcriptional regulators) may be involved in lipid transports (Cheigh et al. 2010; Joon et al. 2010). Casali and Riley (2007) also observed that *mce*1 operon can express independently. Mce1R may express under various negative regulators. Similar observations were also reported by Joon et al. (2010), who strongly supported the existence of two promoters for MTB *mce*1 that could potentially differentiate different functions of one operon. The *mce*1 operon is not the same as the other three *mce* operons, having a *Rv0166* gene (*fadD5*), which is putatively involved in fatty acid catabolism (Joon et al. 2010). The *fadD*5 gene, a fatty acid CoA synthetizer, may be involved in recycling mycolic acids from dying MTB inside granulomas (Cheigh et al. 2010). Dunphy et al. (2010) have shown in their study that mice infected with a MTB mutant in *fadD5* gene, survived longer than those infected with the wild-type strains. They also reported that MTB disrupted in *fadD5* gene is diminished in growth in minimal medium supplied with only mycolic acids (Dunphy et al. 2010).

### 2.2. Mammalian cell entry 2 (mce2) operon

The *mce*2 operon of MTB-encoded proteins which showed highest amino acid identity with *mce*1 operon-encoded proteins (Chitale et al. 2001; Kumar et al. 2003; Ahmad et al. 2005). The *mce*2 operons are present in all *M. avium*, *M. bovis* and *M. smegmatis* species (Haile et al. 2002). The arrangement of *mce*2 operon is different from other three *mce* operons, having an Rv0590A gene fragment between *mce*2B and *mce*2C (Zhang and Xie 2011). However, information about *mce*2 operon-encoded proteins is scanty; Mce2A protein appears to have a distinct role from other *mce* operon-encoded proteins (Uchiya et al. 2013). Mce2 proteins may be involved in the sulfalipids (SL) metabolism and importation during MTB infection (Marjanovic et al. 2011).

Okamoto and colleagues (2006) found that the MTB *mce*2 operon mutants may have an imperfection in the catabolism of cell wall SL, and the accumulation of SL molecules in these mutants may reduce the granuloma formation and inhibit the macrophage activation. Marjanovic et al. (2011) reported that the MTB, with the activation of *mce*2 operon, facilitate the catabolism of SL, remodel architecture of the Mycobacterial cell wall in response to the host immune system, and promote the long-term survival of MTB during infection. Marjanovic et al. (2010) also showed that MTB H37Rv disrupted in *mce*2 gene leads to attenuation in the mouse model of tuberculosis. They also objected that deletion of *mce*2 gene does not affect the Mycobacterial viability *in vitro*. In 2005, Kumar et al. found that the *mce*2 operon could be expressed under all study conditions and that the knock-out of the *mce*2 operon may generate a potential vaccine strain of *M. bovis*. In addition, expression of *mce*2 gene is essential for Mycobacterial growth and might be involved in the latency of these bacteria (Zhang and Xie 2011). In this respect, we can hypothesize that each *mce* operon is selectively expressed under a particular condition during host infection.

### 2.3. Mammalian cell entry 3 (mce3) operon

The *mce*3 operon is not present in MAP, *M. bovis* BCG, *M. smegmatis*, *M*. *microti* and *M. leprae* (Ahmad et al. 2004; Gioffre et al. 2005; Zhang and Xie 2011). Therefore, it was suspected that *mce*3 operon deletion in these bacterial species might contribute to differences in virulence and/or bacterial host range (Bakshi et al. 2005). The absence of *mce*3 within these bacterial species has made this gene an interesting diagnostic candidate (Mitra et al. 2005). Mce3A and Mce3E proteins like Mce1A are also involved in uptake and survival of Mycobacteria (Uchiya et al. 2013). El-Shazly et al. (2007) purified recombinant Mce3A and lipoprotein LprM (Mce3E) from *E. coli* and reported that Mce3A facilitated the internalization and uptake of latex beads by HeLa cells.

Expression studies also revealed that the purified recombinant Mce3A, 3 D and 3E (LprM) protein expression have the ability to elicit antibody responses during MTB infection in human beings (Ahmad et al. 2004; Zhang and Xie 2011). The *mce*3 operon genes are regulated by *mce3R* (*Rv1963*) gene, a tetR transcriptional regulator gene, which regulates the expression of genes which are involved in the metabolism of lipids such as *Ino1* and *Fad*A4 (Santangelo et al. 2008; Marjanovic et al. 2010; Forrellad et al. 2014). These two genes are involved in phosphatidylinositol biosynthetic pathways and lipid degradation, respectively (Santangelo et al. 2008). Furthermore, bioinformatics evidence suggested that Mce3A, 3B, 3C, 3D, 3E and 3F are similar with the other three *mce* operons-encoded proteins in 31–46% of amino acid composition (Ahmad et al. 2005). So the *mce*3 operons relate to the virulence of pathogenic Mycobacteria.

### 2.4. Mammalian cell entry 4 (mce4) operon

The *mce*4 operon is present in most of the Mycobacterial species. It is expressed in the stationary phase of Mycobacterial growth culture or in mammalian hosts (Saini et al. 2008). The *mce*4 operons showed a high degree of conservation in different Mycobacterial species (Haile et al. 2002; Mitra et al. 2005; Timms et al. 2015). It has been shown that Mce4A protein promotes invasion of nonpathogenic *E. coli* strains into non-phagocytic HeLa cells (Saini et al. 2008; Zhang and Xie 2011). Xu et al. (2007) suggested that Mce4A might be a virulence factor which significantly inhibits alveolar macrophage activity. Therefore, deletion of *mce*4 operon attenuates MTB virulence in infected macrophages (Zhang and Xie 2011; Khan et al. 2016). In addition, the Mce4F (Rv3494c) was predicted as Mycobacterial virulence factor which could play a vital role in host cell invasion and could be related to infection adaptation (Rodriguez et al. 2015). The Mce4 proteins are also involved in cholesterol uptake, which is an essential carbon and energy source for Mycobacteria for its prolonged existence in host cells (Pandey and Sassetti 2008; Cheigh et al. 2010; Joon et al. 2010; Klepp et al. 2012; Uchiya et al. 2013). The *mce*4 operon is regulated by KstRregulator, which is involved in fatty acid catabolism (Kendall et al. 2007; Zhang and Xie 2011). Thus, the evidence points to the Mce4 family proteins as of importance for Mycobacterial pathogenesis due to their roles in cholesterol transport with cholesterol being an important nutrient during the Mycobacterial infection (Xu et al. 2007; Mohn et al. 2008; Rathor et al. 2013; Perkowski et al. 2016).

## 3. Distribution of *mce* operons appearing among bacteria

The genus Mycobacterium constitutes a large group of facultative and obligate pathogenic Mycobacteria, e.g. MTB, *M. avium* subsp. *avium*, *M. ulcerans*, *M. leprae* and MAP, causing major diseases in human beings and animals (Haile et al. 2002; Tortoli et al. 2017; Gupta et al. 2018). The homologous regions of *mce* gene families have been reported to be widely distributed in different Mycobacterial species, even in nonpathogenic Mycobacteria (Hemati et al., 2018; Haile et al. 2002; Gioffre et al. 2005; Saini et al. 2008). Parker et al. (1995) reported for the first time the presence of a conserved cellular entry factor, *mce* genes, in Mycobacteria other than the MTB, such as *M. intracellulare*, *M. avium* and *M. scrofulaceum* complex. Casali and Riley (2007) suggested that MTB *mce-*like operons (including six *mce* genes and two *yrbE*) existed within all Mycobacterium species and in five other *Actinomycetales* genera.

Notably, the *mce* loci are present in diverse mycolic acid bacteria which have hydrophobic and thick cell walls, including MTB*mce*, *M. avium* (MA*mce*), MAP (MAP*mce*) *M. bovis* (MB*mce*) and *M. smegmatis* (MS*mce*) and may be found in other species, such as *Nocardia*, *Rhodococcus*, *Janibacter*, *Amycolatopsis*, *Nocardiodes* and *Streptomyces* (Chitale et al. 2001; Haile et al. 2002; Kumar et al. 2003; Casali and Riley 2007; Mohn et al. 2008). *M. smegmatis* and MAP have two copies of the *mce*5 operon; *N. farcinica* and MAP have two copies of the *mce*7 operon; *N. farcinica* possess two copies of the *mce*8 operon and *Streptomyces* has a cluster of *mce*6 operons (Casali and Riley 2007). The *mce*7 operons have a single *mas* gene, the *mce*6 operons of *S. avermilitis* and *N. farcinica* have two copies of *mas* genes, and the *S. coelicolor* operon has four copies of *mas* genes, which all are encoded downstream of *mce* operons (Casali and Riley 2007).

However, the number of *mce* operons varies between Mycobacterial species. For example, the fast-growing Mycobacteria contain the most in contrast to the slow-growing and host-specialized species have less operons, and the obligate intracellular Mycobacteria such as *M. leprae*, have only a single *mce* operon (Miller et al. 2004). Comparison of the *mce* operons encoded in some *Actinomycetales* revealed that these contain an extra *mkl* gene, which encodes an ATPase component resembling those in the ATP-binding cassette (ABC)-transporter system (Casali and Riley 2007). Further studies showed expression of the *mce* genes during steroid and cholesterol metabolism in the Rhodococcal species, with the *mce*4 operon encoding a steroid transporter gene (Kumar et al. 2003; Van der Geize et al. 2007). *Mycobacterium indicus pranii*, an opportunistic pathogen of the MAC family, has extra Mce-related proteins that are common among all the Mycobacterium species except for *M. leprae* and *M. bovis* (Singh et al. 2014b). Sato et al. (2007) have shown that the *mce*1A gene (ML2589) products can mediate entry of *M. leprae* into epithelial cells of the host respiratory tract, whereas anti-Mce1A antibodies can prevent bacterial internalization by epithelial cells. Garcia-Fernández et al. (2017) found that *M. smegmatis* contains 6 *mce* operons that encode ABC-like transporter systems, which are involved in sterol uptake. The *mce*3, *mce*4 and *mce*7 operons of *M. smegmatis* possess the same organization found in *mce* operons of MTB. MS*mce1* operon differs from MTB*mce* operons in having two additional *mas* genes (*MSMEG_5902* and *MSMEG_5893*), whereas MS*mce*5A and MS*mce*5B operons have insertions between the *mce* genes (Garcia-Fernández et al. 2017). The presence of Mce proteins in nonpathogenic Mycobacteria implies their role in mechanisms other than virulence.

Some Gram-negative bacteria additionally contain homologous regions of *mce* gene family, which encode an ABC-transporter-like system, which may be associated with remodeling the bacterial cell envelope (Casali and Riley 2007). In these bacteria, *mce* homologue operons always have the orthologuos of *mkl* genes (Wolf et al. 2001). Many *Proteobacteria* species possess *mce* (PqiB proteins) genes that are analogous to the *mce* complex of *Acinomycetales* (Casali and Riley 2007). Of note, *mce*-associated ATPase of *Pseudomonas putida* make the cells sensitive to toluene (Kim et al. 1998).

In *Neisseria meningitidis* the *glt*T gene is a *mce*-like operon, that is expressed only in invasive hypervirulent isolates (Pagliarulo et al. 2004). Clark et al. (2013) suggested that the deletion of *mce* operon in saprophyte Streptomyces species may have serious effects on bacterial long-term survival in soil environment. More recently, Mce surface protein was described in *Leptospira* species as a novel virulence factor which could mediate the attachment of *L. interrogans* to human cell receptors and are responsible for adherence and invasion mechanisms (Cosate et al. 2016). The presence of *mce* genes, in both Gram-negative bacteria and *Actinomycetales*, affects characteristics of the cell membrane and virulence of the pathogenic species.

## 4. *Mce* operon in MAP

Homologous regions of *mce* gene family have been demonstrated to be present in all of the MAP isolates (Motiwala et al. 2006). The *mce* gene is placed in the outer membrane of MAP (Hemati et al., 2018; Li et al. 2005; Cangelosi et al. 2006). Mce proteins encoded by MB*mce* and MA*mce* operons showed 99.6–100% and 56.2–85.5% homology, respectively, with the respective MTB*mce* proteins (Haile et al. 2002). However, the functions of Mce protein family are not yet been clearly understood in other Mycobacteria (Klepp et al. 2012). In the MAP type K-10 reference genome, the *mce* genes are present in 8 separate clusters containing 6–10 ORFs (Casali and Riley 2007; Xu et al. 2007; Paustian et al. 2008; Castellanos et al. 2009). On the basis of *mce* operons MAP differs from MTB, as MAP has eight-*mce* operons (MAP*mce*1, 2, 3, 4, 5, 5, 7 and 7) instead of four *mce* operons (MTB*mce*1–4) that are present in MTB (Paustian et al. 2008; Castellanos et al. 2009). MAP also possesses two copies of each of the *mce*5 and *mce*7 operons (Casali and Riley 2007).

Individual cluster of *mce* genes in the MAP genome was supposed to encode specific control mechanisms of adaptations that contributed toward entry and survival in different hosts or diverse environments (Zhang and Xie 2011). Timms et al. (2015) reported the missing of conserved hypothetical integral membrane protein *yrb*E3B and *mce*3 operon in MAP K10. They reported that MAP*mce*3 mutant strain grew slower than the parental strain, thus providing a possible explanation for the longer doubling time of MAP. The sequences of the *yrb*E genes associated with *mce* genes of MTB and MAP are listed in Table 3.

**Table 3.** Classification of MAP and MTB H37Rv *yrbE* and *mce* genes.

Prefix^a^	*yrbE1A*	*yrbE1B*	*mce1A*	*mce1B*	*mce1C*	*mce1D*	*mce1E*	*mce1F*
MAP	3602	3603	3604	3605	3606	3607	3608	3609
MTB	0167	0168	0169	0170	0171	0172	0173	0174
	*yrbE2A*	*yrbE2B*	*mce2A*	*mce2B*	*mce2C*	*mce2D*	*mce2E*	*mce2F*
MAP	4082	4083	4084	4085	4086	4087	4088	4089
MTB	0587	0588	0589	0590	0591	0592	0593	0594
	*yrbE3A*	*yrbE3B*	*mce3A*	*mce3B*	*mce3C*	*mce3D*	*mce3E*	*mce3F*
MAP	2117^b^	2117^b^.1^c^	2116^b^	2115^b^	2114^b^	2113^b^	2112^b^	2111^b^
MTB	1964	1965	1966	1967	1968	1969	1970	1971
	*yrbE4A*	*yrbE4B*	*mce4A*	*mce4B*	*mce4C*	*mce4D*	*mce4E*	*mce4F*
MAP	0562	0563	0564	0565	0566	0567	0568	0569
MTB	3451^b^	3450^b^	3499^b^	3498^b^	3497^b^	3496^b^	3495^b^	3494^b^
	*yrbE5A*	*yrbE5B*	*mce5A*	*mce5B*	*mce5C*	*mce5D*	*mce5E*	*mce5F*
MAP	–	–	2189	2190	2191	2192	2193	2194
MTB	–	–	–	–	–	–	–	–
	*yrbE6A*	*yrbE6B*	*mce6A*	*mce6B*	*mce6C*	*mce6D*	*mce6E*	*mce6F*
MAP	–	–	–	–	–	–	–	–
MTB	–	–	–	–	–	–	–	–
	*yrbE7A*	*yrbE7B*	*mce7A*	*mce7B*	*mce7C*	*mce7D*	*mce7E*	*mce7F*
MAP	1849	1850	1851	1852	1853	1854	1855	1856
MTB	–	–	–	–	–	–	–	–
	*yrbE8A*	*yrbE8B*	*mce8A*	*mce8B*	*mce8C*	*mce8D*	*mce8E*	*mce8F*
MAP	***–***	***–***	***–***	***–***	***–***	***–***	***–***	***–***
MTB	***–***	***–***	***–***	***–***	***–***	***–***	***–***	***–***

^a^Organism specific gene number prefix: MAP; MTB H37Rv.

^b^Orthologous sequence present, but Open Reading Frame (ORF) annotated in reverse direction.

^c^Orthologous sequence present, but not annotated. ORF extends ∼400 bp at 5′end.

Whole-genome DNA microarray representing MAP revealed that there are 14 large sequence polymorphisms (Motiwala et al. 2006). LSPP12 was the most widely conserved MAP*-*specific region that included a cluster of six homologous of the *mce*-family (MAP*2189*-MAP*2194*), which is involved in lipid metabolism (Hemati et al., 2018; Semret et al. 2005; Alexander et al. 2009). In MAP, *mas* homologous genes were located in pairs (MAP0750-51c, MAP0767-68c) both downstream and upstream of the MAP*mce*5 operon (Casali and Riley 2007). These genes have been identified as important for MAP invasion, survival and virulence (Semret et al. 2005). Large sequence polymorphisms (LSPs) having diagnostic importance and in total 14 LSPs have been identified till to date, whereas LSP11 was absent in the MAP isolates, which comprised part of a *mce*2 operon (Motiwala et al. 2006). The loss of *mce*2 or, *mce*3, genes in the most pathogenic MAP isolates along with the deletion of *mce*3 from virulent *M. bovis* together prove either *mce*2 and *mce*3 operons to act as virulence factors (Semret et al. 2004). Timms and colleagues (2015) observed the gap between *mycobactin* A and *mycobactin* J (*lip*K) genes in all MAP genomes containing at least one *mce* operon 8.7 kB downstream from *mycobactin* A gene and a link between the *mce* operons and the mycobactin cluster genes. Studies on the importance of the close proximity of this operon with mycobactin cluster are currently underway.

## 5. Mechanisms of function of *mce* genes

Mechanism and function of *mce* genes is not very clear yet, but Mycobacterial species not having *mce* genes cannot enter the host cell and thereby the severity of infection could be reduced (Castellanos et al. 2012). Several observations strengthen this hypothesis: gene knockouts of *mce*1–*mce*3 and *mce*4 in MTB and *M. bovis* (BCG) cause attenuation of these strains in mouse models (Castellanos et al. 2012); inactivation of *mce* genes could reduce the ability of MTB to invade and persist in the host cells (Gioffre et al. 2005); *mce*3 operon mutant of MTB was attenuated in mice (Senaratne et al. 2008); the *mce*4 operon mutant of MTB have shown growth defect and significantly reduced bacterial survival in infected mice (Saini et al. 2008). Most recently, Zhang et al. (2017) have shown that Mce3C as MTB surface protein could interact with β2 integrin and cause clustering at Mycobacterial entry site. In the host mammalian cells, interaction between adhesion proteins such as integrin and their ligand is essential for cell proliferation and growth, thus, the interaction between Mce proteins and integrin may be involved in an adhesion-dependent mechanism (Simoes et al. 2005).

Kumar et al. (2005) suggested that invasion of the host cell is not the only function of *mce* operons. Mce-family proteins may also have a role in pathogenesis by inhibiting alveolar macrophage activity or eliciting immune response from the host, may serve as lipid transporters by analogy to ABC-transporters and also can be related to granuloma formation and long-term survival of Mycobacteria within the host cells with all above attributes playing a very important role in Mycobacterial virulence (Mohn et al. 2008; Marjanovic et al. 2010; Rathor et al. 2013; Rodriguez et al. 2015; Perkowski et al. 2016). A previous study has shown that *mce* genes may have a role in the maintenance of cell surface properties in Mycobacteria and can be contributed to the cell envelope production (Klepp et al. 2012). Furthermore, some Mce proteins of the Mycobacterial membrane may contribute to the creation of beta barrel proteins serving as channels (by six Mce proteins with similarity to substrate binding proteins and two YrbE proteins with similarity to ABC-permeases) and may function as ABC-transport systems (Li et al. 2005; Cangelosi et al. 2006; Pandey and Sassetti 2008; Paustian et al. 2008; Perkowski et al. 2016). Arrangements of Mce proteins are structurally similar to ABC-transporters and due to the cell surface location of Mce proteins; it has been suggested that they may play a role in cell invasion of cholesterol-rich regions and immuno-modulation (Lamont et al. 2014; Khan et al. 2016). Mohn et al. (2008) determined that in *Rhodococcus jostii* RHA1 (with 2 *mce* operons), *mce*4 operon encodes an ATP-dependent steroid transporter that was essential for bacterial growth on media containing a range of sterols as the only carbon source.

This novel type of ABC-transporter system encoded by *mce* loci is believed to be involved in both import of fatty acids as a source of nutritional carbons and the export of a variety of lipid virulence factors during Mycobacterial growth (Wang et al. 2007). Just like ABC-type transporters, Mce transporter system can specifically bind with small lipid molecular compounds (Zhang and Xie 2011). The nature of their substrates has only been revealed in the case of the Mce4 proteins with cholesterol as one potential substrate (Forrellad et al. 2014). Pajon et al. (2006) found that eight-Mce proteins of MTB could help piercing of the outer lipid layer and could form a channel through this lipid bilayer. Therefore, it is possible that these Mce proteins may be more important for the transport of solutes through hydrophobic barriers such as host cell membranes or the Mycobacterial envelope (Joshi et al. 2006). The *mce*4 operon of *R. equi* encodes an active system for steroid uptake, such as cholesterol, 5-α-cholestanol and β-sitosterol (Mohn et al. 2008). This hypothesis is supported by the recent finding that the deletion of *mce*4 operon was responsible for the cholesterol uptake failure in the *mce*4-deficient strain (Kelpp et al. 2013). Saprophytic Mycobacteria with steroid uptake activity might be able to detect the presence of abundant steroid substrates in the nature.

Another possibility is that the ability of Mce4 proteins to bind to cholesterol-rich areas of the cell membrane may play a vital role in the pathogenesis of Mycobacteria by localizing the Mycobacterium, modifying the host cell membrane, facilitating host cell entry and blocking the normal phagosome maturation or eliciting an important immune response from the host (Keown et al. 2012). It has been demonstrated that the *mce*4 operons are involved in the cholesterol uptake in MTB (Pandey and Sassetti 2008), *R. equi* (van der Geize et al. 2007), *M. smegmatis* (Klepp et al. 2012) and *R. jostii* RHA1 (Mohn et al. 2008). Remarkably, the uptake of this steroid by Mce4A protein in MTB has been linked to its long-term survival ability in the host (khan et al. 2016).

Besides, Mce-family proteins as cell surface proteins are recognized by the immune system of the host in the involvement of Mycobacterial species virulence (Ghosh et al. 2012). The functional importance of these highly antigenic *mce* operons is illustrated by their differential expression profile in bacilli under different culture conditions and during infection (Ahmad et al. 2004; Joon et al. 2010; Pasricha et al. 2011). The expression of *mce* operons in Mycobacteria may be modulated in response to stress conditions and nutritional status, however, the extra-cellular signals required for *mce* expression are not known yet (Zhu et al. 2008).

## 6. Conclusions

In conclusion, the detection of Mce proteins with high immunogenicity can be a big step in the early diagnosis of Mycobacterial diseases. Surface location of the *mce* proteins makes them interesting early diagnostic markers. Taken together, the functional importance of *mce* operons invites further studies, however work done so far has shown that there are immune-dominant epitopes within *mce* genes, suggesting that these could potentially be exploited as a source of antigenic proteins for the diagnosis of all the Mycobacterial species notably MAP.
